# Epidemiological analysis of maternal deaths in Hunan province in China between 2009 and 2014

**DOI:** 10.1371/journal.pone.0207920

**Published:** 2018-11-26

**Authors:** Xiong Lili, He Jian, Zeng Mengjun, Wu Yinglan, Xie Donghua, Wang Aihua, Kong Fanjuan, Wang Hua, Liu Zhiyu

**Affiliations:** 1 Department of Information Management, Hunan Province Maternal and Children Health Care Hospital, Changsha, Hunan, China; 2 Department of Maternal Healthcare Department, Hunan Province Maternal and Children Health Care Hospital, Changsha, Hunan, China; Aga Khan University, PAKISTAN

## Abstract

**Background:**

The control of maternal deaths continues to be a significant public health issue and commands an enormous amount of attention, especially under the future family planning policy. Here, we describe the epidemiology and trends of maternal deaths in Hunan province, and give several policy implications.

**Methods:**

Maternal deaths in Hunan province between 2009 and 2014 were retrospectively reviewed and analyzed. Cochran-Armitage trend test was used to assess the time trends of maternal mortality rates. Binary logistic regression analyses were undertaken to identify the factors that were associated with unavoidable maternal deaths.

**Results:**

In total, there were 987 maternal deaths, with the overall MMR declining by 45.24%. The most common causes of maternal death during this period were pregnancy complications (28.37%), obstetric hemorrhage (25.33%), and amniotic fluid embolism (15.70%). Obstetric hemorrhage (28.14%) was higher in rural areas, while pregnancy complications were higher (29.27%) in urban areas. In all, 627 (63.5%) deaths were avoidable. The risk factors associated with unavoidable maternal deaths was above 35 years *(aOR* = 1.80 95%*CI*: 1.27–2.55), without prenatal examination (*aOR* = 8.97 95%*CI*: 1.11–7.78), low household incomes (*aOR* = 1.15 95%*CI*: 1.02–1.29), without adopting the new way to deliver (*aOR* = 5.15 95%*CI*: 3.20–8.31), and death location (*aOR* = 1.09 95%*CI*: 1.02–1.18). The most frequent and important factors associated with avoidable deaths was improper knowledge and skills of the county medical institutions.

**Conclusions:**

Moderate progress was made in reducing the MMR in Hunan province. The government should aim to improve the basic midwifery skills in rural areas and the obstetric emergency rescue service for critically ill pregnant women in urban areas, and strengthen training to improve knowledge and skills in medical institutions in counties.

## Introduction

Every maternal death represents not just the loss of a woman’s life, but the impact of that loss upon her family and the community. Consequently, for each country, maternal mortality is considered a sentinel event, reflecting the state of maternal health, as well as the general state of health care. The maternal mortality ratio (MMR) that expressed as the number of deaths per 100,000 live births is one of the most important and globally-recognized indicators for measuring the state of a country’s economy, culture, and healthcare system. The MMR in developing regions was 14 times higher than in developed regions. Low- and middle-income countries collectively represent the source of 99% of global maternal deaths. China has the largest population in the world. In 2000, China recorded 11,000 maternal deaths, ranking the country in within the top 13 countries with the highest number of maternal deaths [1]. China endorsed the United Nations MDGs in 2000, which included a reduction of the MMR by 2015 from 89 to 22 between 1990 and 2015. However, the MMR in China decreased to 20 in 2015 [2].

Although the era of MDGs has come to an end, in January 2013, the Ending Preventable Maternal Mortality Working Group, led by the WHO with support from partner organizations, set new targets for maternal health and survival from 2015 to 2030. Two of these goals are that by 2030, every country should reduce its MMR by at least two thirds from their 2010 baseline and all countries are tasked with achieving equity in MMR among sub-populations [3]. Although substantial progress has been made in the control of maternal mortality in China, progress has generally been slow and shown obvious disparities across regions [4, 5]. Generally, a decreasing trend in MMR was recorded in China, with a reduction of 38.2%, 59.7%, and 75.6%, respectively, in coastal, inland and remote areas. Data also showed a 32.4% reduction in urban areas of China and a 71.0% reduction in rural areas between 2000 and 2015 [2]. What’s more, implementation of the universal two-child policy in 2016 would make maternal death control more important.

Given the area and diversity of China, reducing maternal deaths and achieving equity across the whole of China under the same policies is a significant challenge. Thus, provincial maternal mortality surveillance systems (PMMSS) have been set up in nearly all of the provinces in China in order to monitor local maternal mortality levels and to provide policy suggestions for promoting maternal and child healthcare (MCH) suitable to local situations. For example, maternal mortality in Shanghai decreased steadily from 2000 to 2009, from 21.2 to 9.61 between 2000 and 2009, comparable to the level of many developed countries [6]. In addition, the total MMR in Henan Province declined by 78.4%, from 80.1 to 17.3 between 1996 and 2009 [7]. These data were all monitored in the system.

While there have been numerous studies describing maternal mortality in China, to which we refer later, there are no publications which specifically focus on Hunan Province, and so far, there are seldom report based on the whole province except Henan province [7]. The goal of this present study was therefore to determine the epidemiology and trends in maternal deaths, from surveillance data obtained from 2009 to 2014 in Hunan province. We hope that this study will provide a timely policy reference base with which to support the universal two-child policy and to create regulations intended to reduce maternal deaths. The epidemiological results of our study will also assist in the development of national maternal death prevention strategies in China.

## Data and methods

### Data sources

The maternal death case data used in this study were obtained from PMMSS, a well-established population-based maternal death registry system covering 14 cities from all 123 counties in Hunan province. According to the WHO, maternal death was defined as the death of a woman while pregnant, or within 42 days of the termination of pregnancy, irrespective of the duration and site of the pregnancy, from any cause related to, or aggravated by, the pregnancy or its management but not from accidental or incidental causes [8]. Cases of maternal death in patients who resided in Hunan province for at least one year were the surveillance subjects herein.

Live births from 2009 to 2014 were obtained from the Hunan province annual report. MMR was calculated by comparing the total number of maternal deaths with the total number of live births during the same year, and all MMRs were expressed as the number of deaths per 100,000 live births. The study protocol was reviewed and approved by the Institutional Review Board at Hunan province maternal and children health care hospital.

Hunan province was located in central China, covers 21.18 km^2^, and had a population of 71.47 million people, with 14 cities and 123 counties. The PMMSS system was established in October 1995 and was fully implemented in January 1996, and has now expanded to cover the entire province.

### Maternal death review and ascertainment of outcome

**Fig 1:**
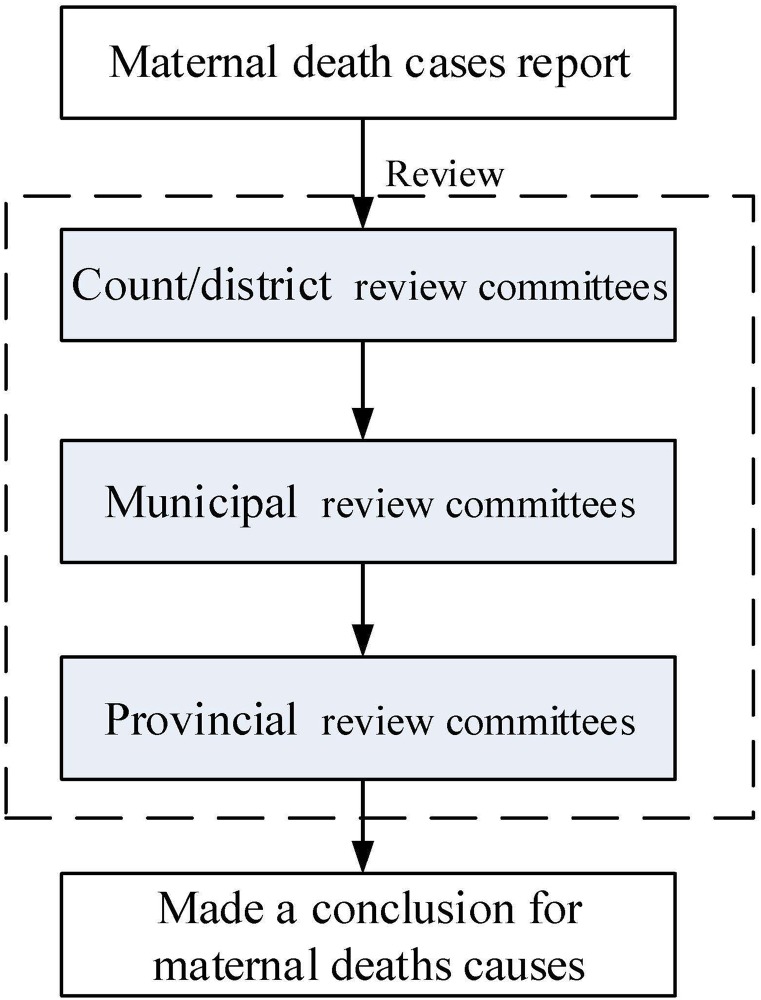
The flow chart of maternal deaths review process in Hunan province, China.

According to the WHO recommendations [9], each case of maternal death was reviewed firstly by the review committees at the county/district, then by the review committees at the municipal medical institutions, and finally at the provincial medical institutions, which ensured that all maternal death data were assessed by experts every six months in order to determine the causes of death and establish preventability, as well as to identify any associated factors. Death data were evaluated very carefully and all uncertain data were reinvestigated to ensure that the causes of death were reliable. In the end, the maternal death review conclusion was made by the provincial committees, especially when multiple reviewer conclusions made by the review committees of municipal and provincial medical institutions assigned different cause of death on a particular death (Fig 1). Direct death causes referred to obstetric complications occurred during pregnancy, at delivery, or postpartum, such as abortion, ectopic pregnancy, postpartum haemorrhage, pre-eclampsia, and puerperal infection. Indirect causes of maternal death refer to a previously existing disease or a disease that developed during pregnancy unrelated to an obstetric event but was aggravated by physiological changes of pregnancy. Avoidable maternal death can refer to the institution or provider, and patient factors which may have ultimately prevented a condition from causing death or severe morbidity. The factors associated with avoidable maternal death involved knowledge/skills, attitude, resources and management in individuals/families, health care facilities and community services [10]. Every case was reviewed to determine whether it was avoidable.

### Quality control

County/district and municipal MCH hospitals shall organize two comprehensive quality inspections every year. The provincial MCH hospital shall conduct a yearly survey of quality inspection. Those that fail to meet the requirement fully do the survey for the omission report of maternal deaths. The content of quality control was the underreport rate of the live births and the maternal deaths, the error rate of the card filling, and the complete rate of the card filling.

### Statistical analysis

Data were exported to Microsoft Excel 2010 and analyzed with SAS Version 9.3. Descriptive statistics, such as the frequencies and percentages of baseline variables were calculated. Maternal cause-specific mortality and region-specific mortality rates per 100,000 live births were also calculated. Time trends in MMR were tested using the Cochran-Armitage trend test. The standard chi-square test was used to compare the distributions of epidemiological, delivery, and maternal care characteristics for unavoidable and avoidable maternal deaths. Binary logistic regression model was used to examine the factors that were associated with unavoidable maternal deaths after selecting the variables into the adjusted model simultaneously, which had statistical significance in simple logistic regression. *P*<0.05 was chosen as the level of statistical significance.

## Results

### MMR in Hunan province

**Fig 2:**
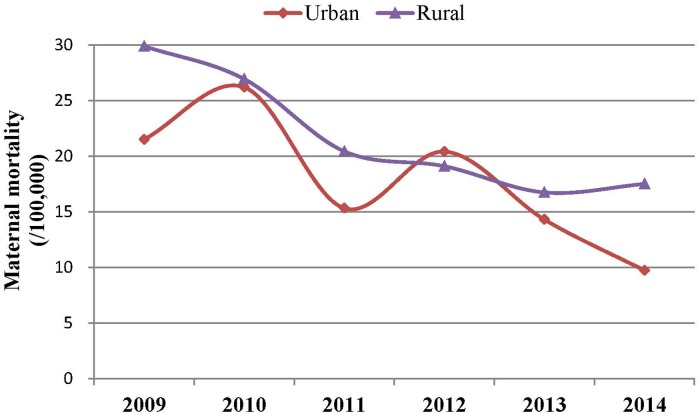
Trends of maternal mortality in Hunan province, China, between 2009 and 2014.

Between 2009 and 2014, there was a total of 987 maternal deaths (20.46 per 100,000 live births), of which 287 (17.89 per 100,000 live births) occurred in urban areas and 700 (21.73 per 100,000 live births) occurred in rural areas. The MMR declined by 45.24% across the whole province, from 27.14 to 14.86. The Cochran–Armitage trend test showed a gradual decline in total MMR with a *Z* value of −6.90 and *P*<0.001 (Table 1, Fig 2).

**Table 1:** Basic information relating to maternal deaths in Hunan province, China, between 2009 and 2014.

Basic information	Area	2009		2010		2011		2012		2013		2014	
		N	%	N	%	N	%	N	%	N	%	N	%
Total live births	Urban	255649	15.93	263350	16.41	267356	16.66	279088	17.39	272312	16.97	266739	16.62
	Rural	525377	16.31	534552	16.60	538004	16.70	559886	17.38	549043	17.05	513835	15.95
Obstetric hemorrhage	Urban	11	20.75	13	24.53	6	11.32	12	22.64	8	15.09	3	5.66
	Rural	37	18.78	48	24.37	38	19.29	31	15.74	18	9.14	25	12.69
Pregnancy-induced hypertension	Urban	4	21.05	6	31.58	2	10.53	4	21.05	0	0.00	3	15.79
	Rural	9	19.15	13	27.66	11	23.40	6	12.77	3	6.38	5	10.64
Pregnancy complications	Urban	11	13.10	22	26.19	14	16.67	14	16.67	14	16.67	9	10.71
	Rural	52	26.53	34	17.35	23	11.73	30	15.31	35	17.86	22	11.22
Amniotic fluid embolism	Urban	14	27.45	6	11.76	8	15.69	12	23.53	7	13.73	4	7.84
	Rural	27	25.96	19	18.27	9	8.65	22	21.15	13	12.50	14	13.46
Others	Urban	15	18.75	22	27.50	11	13.75	15	18.75	10	12.50	7	8.75
	Rural	32	20.51	30	19.23	29	18.59	18	11.54	23	14.74	24	15.38
Total	Urban	55	19.16	69	24.04	41	14.29	57	19.86	39	13.59	26	9.06
	Rural	157	22.43	144	20.57	110	15.71	107	15.29	92	13.14	90	12.86
Obstetrics death causes	Direct	108	21.86	115	23.28	75	15.18	93	18.83	51	10.32	52	10.53
	Indirect	104	21.76	97	20.29	69	14.44	71	14.85	79	16.53	58	12.13
	Unknown	0	0.00	1	6.67	7	46.67	0	0.00	1	6.67	6	40.00
Total maternal deaths		212	21.48	213	21.58	151	15.30	164	16.62	131	13.27	116	11.75

**Fig 3:**
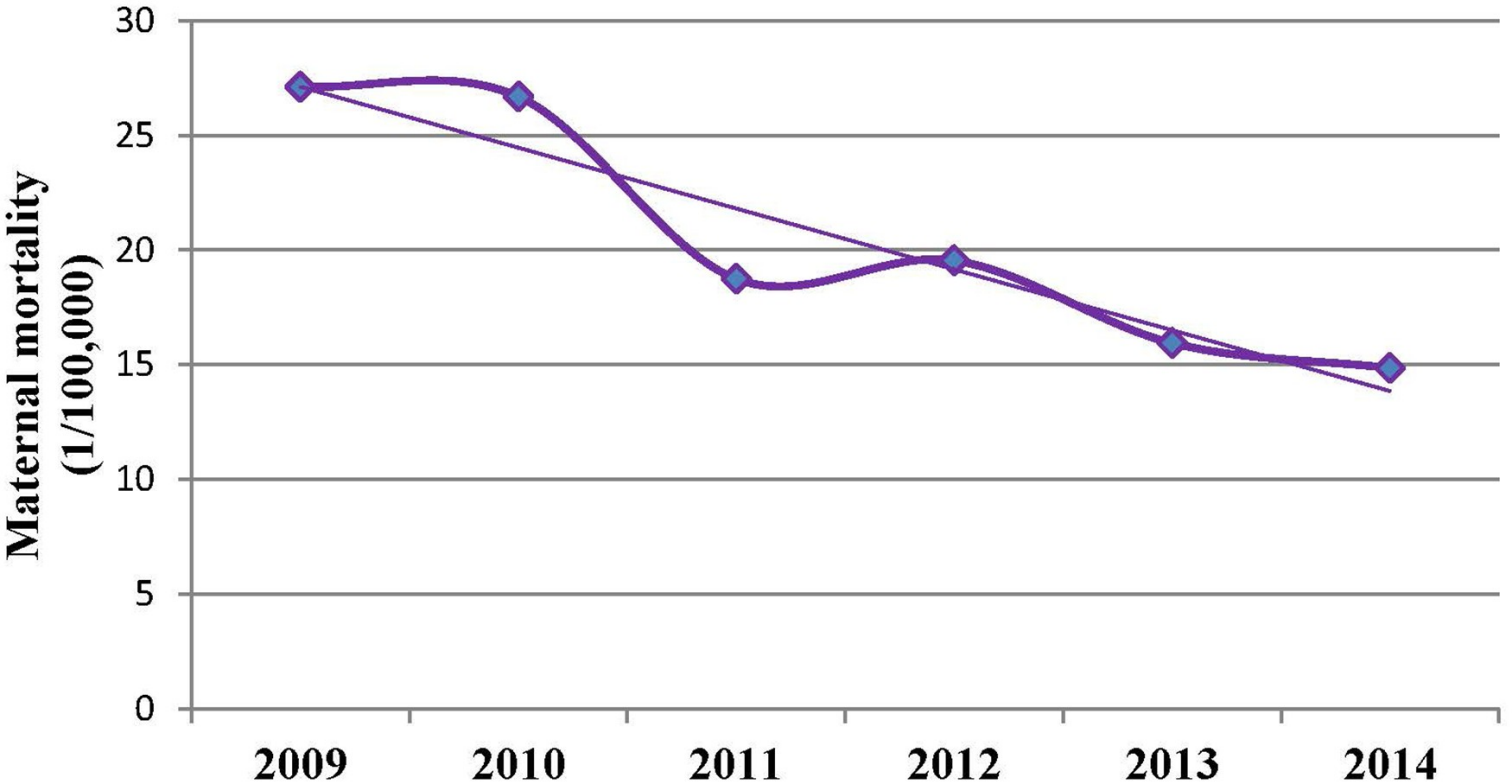
Trends of maternal mortality in urban and rural areas in Hunan province, China, between 2009 and 2014.

Similarly, the MMR decreased by 54.67% in the urban areas: from 21.51 to 9.75 (*Z=*−4.11, *P*<0.001). The corresponding MMR reduction was by 41.37% in the rural areas: from 29.88 to 17.52 (*Z=*−5.54, *P*<0.001). The MMRs in rural areas were significantly higher than that in urban areas in 2009 (*χ*^*2*^ = 4.44, *P* = 0.03) and in 2014 (*χ*^*2*^ = 7.13, *P* = 0.01), but not in any of the other years (*P*>0.05) (Table 1, Fig 3).

### Causes of maternal death

The proportions of maternal deaths due to direct obstetric causes was 50.05% cases (*N* = 494), and 48.43% cases for indirect causes (*N* = 478). 1.52% of cases (*N* = 15) were unable to review the causes of death. The proportion of direct obstetric causes was higher than that of indirect causes from 2009 to 2012, while the opposite was observed from 2013 to 2014 (*χ*^*2*^ = 34.07, *P*<0.01). The leading cause of maternal death was pregnancy complications; MMR was 5.80, accounting for 28.37% of all maternal deaths. The second was obstetric hemorrhage (MMR = 5.18), accounting for 25.33% of all maternal deaths. The third was amniotic fluid embolism (MMR = 2.1) accounting for 15.70% of all maternal deaths. There were 236 deaths (23.91%) which were classified as ‘other causes’.

**Fig 4:**
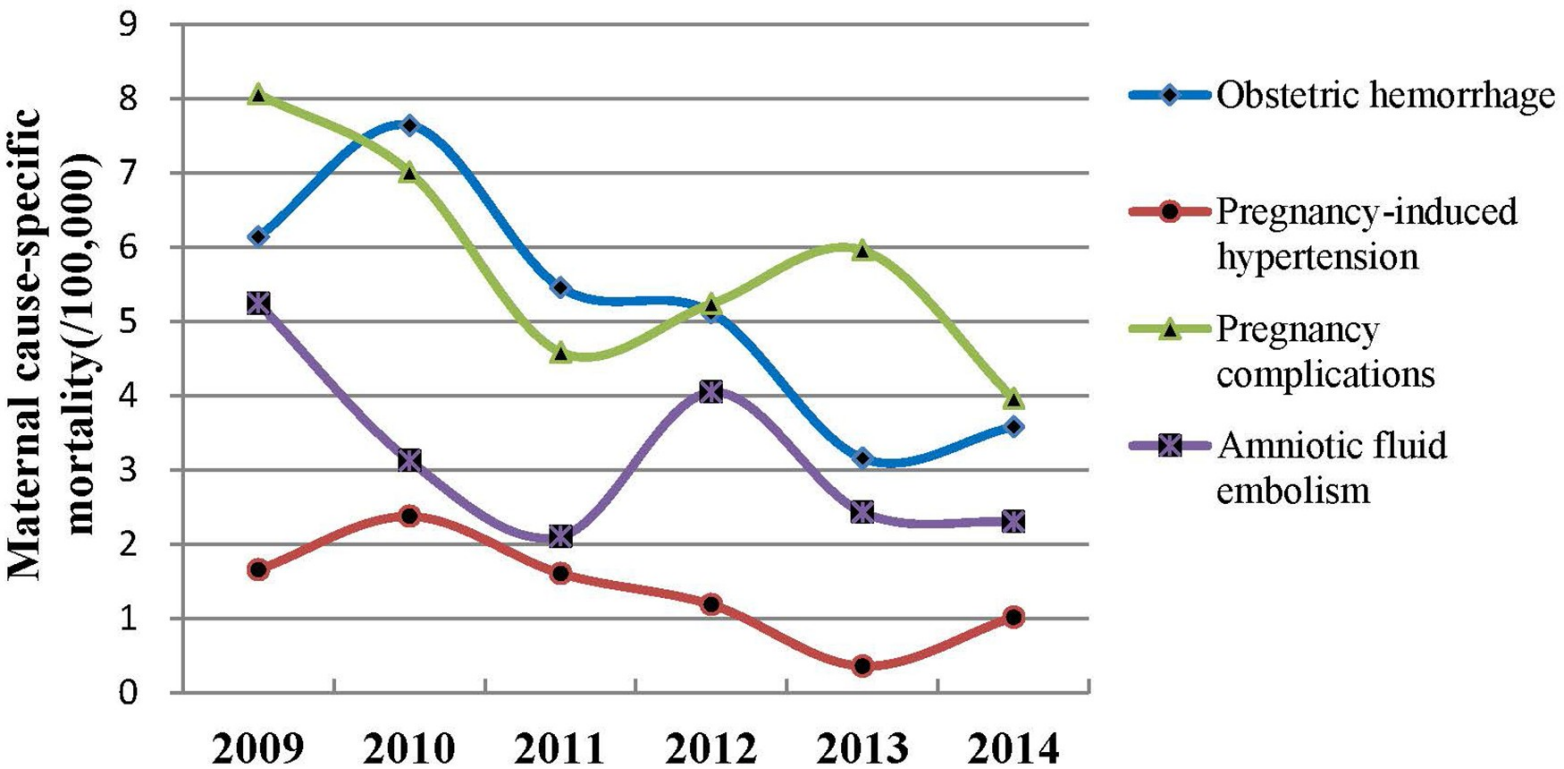
Trends of maternal cause-specific mortality in Hunan province, China, between 2009 and 2014.

Between 2010 and 2011, the leading cause of death was obstetric hemorrhage with an MMR from 7.65 to 5.46, but in the other years the leading cause of death was pregnancy complications with an MMR from 8.07 to 3.97. The MMR of amniotic fluid embolism was declined by 56.07% from 5.25 to 2.31 between 2009 and 2014. The Cochran–Armitage trend test showed a gradual decline in the MMR arising from obstetric hemorrhage with a *Z* value of −3.95 and *P*<0.001. Furthermore, MMR arising from pregnancy complications decreased by 50.77%, and maintained a gradual decline (*Z* = −3.18, *P* = 0.005) (Table 1, Fig 4).

**Fig 5:**
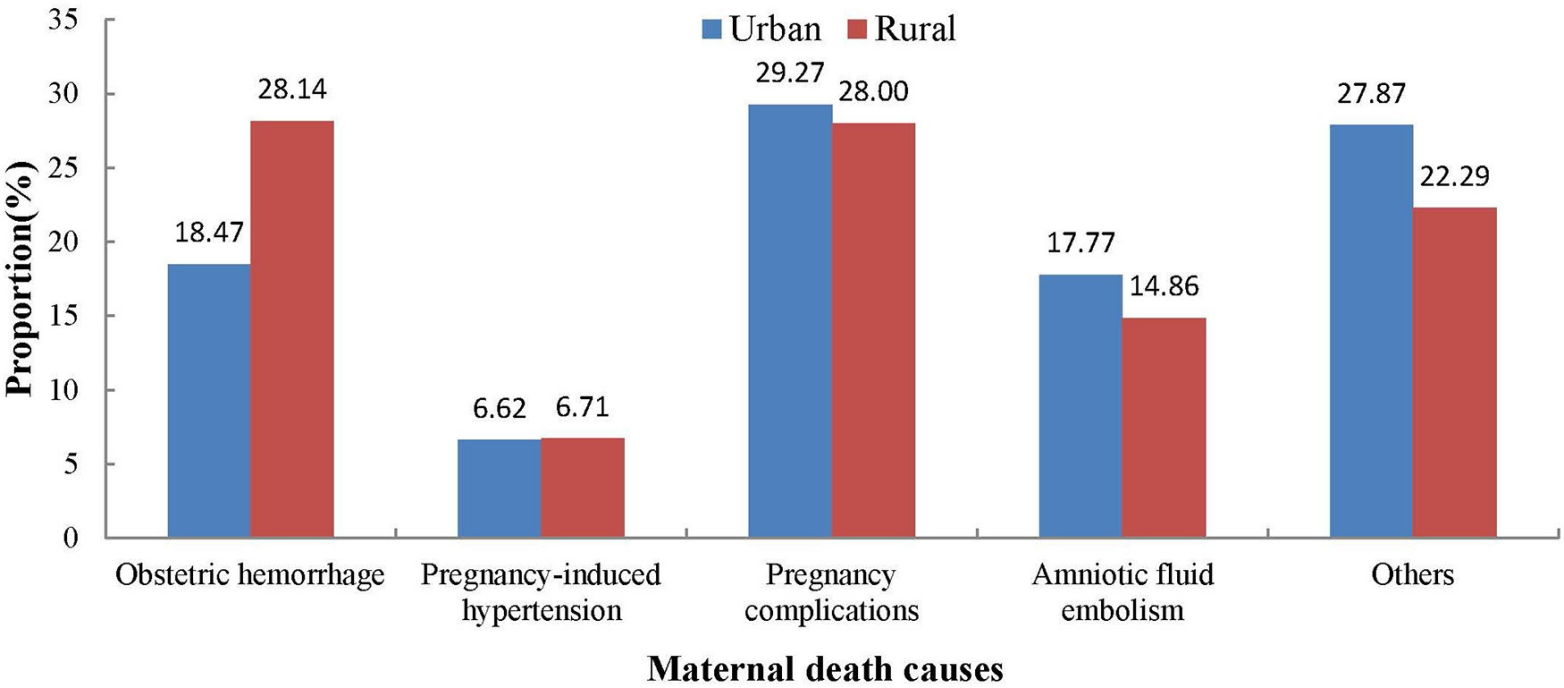
The causes of maternal deaths when compared between urban and rural areas of Hunan province, China, between 2009 and 2014.

Pregnancy complications, with an MMR of 5.24, was the leading cause of maternal death in urban areas, accounting for 29.27%(*N* = 196) in Hunan province between 2009 and 2014, while the leading cause of maternal death in rural areas was obstetric hemorrhage, with an MMR of 6.12, accounting for 28.14% (*N* = 197) (Table 1, Fig 5). The proportions of different causes of maternal death differed significantly between rural and urban areas (*χ*^*2*^ = 8.17, *P* = 0.04).

### Characteristics of maternal death

Epidemiological, delivery, and maternal care characteristics of maternal death cases from 2009 to 2014 in Hunan province are presented in Table 2. More than 72% of deaths (*N* = 715) involved mothers aged between 20 and 34 years. Furthermore, 77.4% (*N* = 763) of maternal death cases had not attended high school, 57.4% (*N* = 567) resided in the mountains, 31.6% (*N* = 312) occurred in the municipal hospital or provincial level medical institutions, 56.4% (*N* = 557) of maternal deaths occurred in the first 7 days after delivery. 35% (*N* = 342) required caesarean section, 9.4% women (*N* = 94) never attended an antenatal care clinic prior to delivery. The variables of age groups, educational level, household incomes per capita, death location, delivery locations, the days from delivery to death, parity, gestational age, mode of delivery, prenatal examination, and prenatal examination times were statistically significant in simple logistic mode that examined the risk factors of unavoidable maternal deaths (Table 2).

**Table 2:** The total number of deaths and the distribution of epidemiological, delivery, and maternal care characteristics factors related to unavoidable and avoidable maternal deaths in Hunan province, China, between 2009 and 2014.

Factors	Total(*N*)	%	Unavoidable	avoidable	Unreviewable	*OR(*95%CI)*
Epidemiological characteristics						
Year			χ2* = 6.26, *P* = 0.282			
2009	212	21.48	64	148	0	0.66(0.41–1.06)
2010	213	21.58	78	135	0	0.88(0.55–1.41)
2011	151	15.3	45	98	8	0.70(0.42–1.18)
2012	164	16.62	62	102	0	0.93(0.57–1.52)
2013	131	13.27	52	77	2	1.03(0.61–1.73)
2014	116	11.75	44	67	5	RE
Age groups			χ2* = 13.94, *P*<0.001			
> = 35	248	25.1	63	180	5	0.85(0.34–2.15)
20–34	715	72.4	275	430	10	1.55(0.64–3.79)
<20	24	2.4	7	17	0	RE
Educational level			χ2* = 17.73, *P* = 0.001			
Illiterate	42	4.3	9	33	0	0.24(0.09–0.61)
Primary school	135	13.7	33	100	2	0.29(0.14–0.59)
Middle school	586	59.4	209	369	8	0.50(0.27–0.91)
High school	177	17.9	70	104	3	0.59(0.31–1.14)
Bachelor or above	47	4.8	24	21	2	RE
Household incomes per capita			χ2* = 18.93, *P*<0.001			
<1000	152	15.4	41	110	1	0.39(0.25–0.62)
1000~	239	24.2	80	157	2	0.53(0.36–0.80)
2000~	251	25.4	80	168	3	0.50(0.33–0.75)
4000~	170	17.2	61	105	4	0.61(0.39–0.94)
8000~	175	17.7	83	87	5	RE
Residence area			χ2* = 2.28, *P* = 0.32			
Plain	253	25.6	96	156	1	0.99(0.66–1.49)
Mountains	567	57.4	188	373	6	0.81(0.56–1.17)
Others	167	16.9	61	98	8	RE
Death locations			χ2* = 52.56, *P*<0.001			
Others /Missing	26	2.6	7	19	0	0.47(0.19–1.15)
In transport	130	13.2	46	83	1	0.71(0.46–1.08)
Home/Village clinic	141	14.3	73	67	1	1.39(0.93–2.07)
Town hospital	88	8.9	16	71	1	0.29(0.16–0.52)
County\district hospital	290	29.4	68	215	7	0.40(0.28–0.57)
Municipal and provincial hospital	312	31.6	135	172	5	RE
Delivery locations			χ2* = 87.65, *P*<0.001			
Others/ Missing	313	31.71	136	172	5	0.62(0.43–0.89)
Home /Village clinic	44	4.46	2	41	1	0.04(0.01–0.16)
Town hospital	131	13.27	17	112	2	0.12(0.07–0.21)
County\district hospital	295	29.89	78	214	3	0.29(0.20–0.42)
Municipal and provincial hospital	204	20.67	112	88	4	RE
The days from delivery to death			χ2* = 67.37, *P*<0.001			
≤1day	253	25.6	38	210	5	0.19(0.13–0.30)
1-7days	304	30.8	104	198	2	0.56(0.40–0.79)
7-42days	158	16	74	81	3	0.98(0.66–1.45)
Missing	272	27.6	129	138	5	RE
Death time			χ2* = 8.69, *P* = 0.07			
0:00–6:00	297	30.1	106	183	8	0.82(0.37–1.78)
6:00–12:00	235	23.8	64	168	3	0.54(0.24–1.19)
12:00–18:00	221	22.4	84	135	2	0.88(0.40–1.94)
18:00–24:00	203	20.6	79	124	0	0.90(0.41–1.99)
missing	31	3.1	12	17	2	RE
Death season			χ2* = 3.13, *P* = 0.36			
Spring	223	23.6	83	137	3	0.86(0.60–1.24)
Summer	227	23	85	139	3	1.10(0.76–1.58)
Autumn	257	26	79	175	3	0.81(0.56–1.17)
Winter	280	28.4	98	176	6	RE
Delivery characteristics						
Parity			χ2* = 38.42, *P*<0.001			
Nulliparous	140	14.18	72	65	3	4.05(2.29–7.16)
1	420	42.55	165	249	6	2.42(1.47–4.00)
2	316	32.02	85	229	2	1.36(0.80–2.29)
≥3	111	11.25	23	84	4	RE
The abortion/induced labor times			χ2* = 5.40, *P* = 0.15			
0	514	52.08	190	313	11	1.70(1.03–2.80)
1	243	24.62	80	163	0	1.37(0.80–2.35)
2	139	14.08	51	84	4	1.70(0.95–3.03)
≥3	91	9.22	24	67	0	RE
Gestational age			χ2* = 39.39, *P*<0.001			
<24 weeks	17	1.72	4	13	0	0.39(0.12–1.23)
24–36 weeks	134	13.58	67	66	1	1.29(0.86–1.94)
≥36 weeks	526	53.29	140	378	8	0.47(0.35–0.63)
Missing	310	31.41	134	170	6	RE
Mode of delivery			χ2* = 55.12, *P*<0.001			
Caesarean section	342	34.65	137	202	3	0.92(0.67–1.26)
Vaginal operative delivery	56	5.67	17	39	0	0.47(0.25–0.87)
Vaginal delivery	319	32.32	63	249	7	0.27(0.19–0.39)
Undelivered	270	27.36	128	137	5	RE
Adopt new delivery way			χ2* = 41.85, *P*<0.001			
Missing	281	28.5	143	132	6	2.36(1.77–3.14)
No	31	3.1	3	27	1	0.24(0.07–0.81)
Yes	675	68.4	210	457	8	RE
Maternal care characteristics						
Prenatal examination			χ2* = 8.82, *P* = 0.003			
Yes	893	90.48	325	553	15	2.18(1.30–3.63)
No/Unknown	94	9.52	20	74	0	RE
Initiation of prenatal care			χ2* = 2.97, *P* = 0.40			
First trimester	771	78.12	280	480	11	1.75(0.47–6.52)
Second trimester	170	17.22	51	115	4	1.33(0.35–5.12)
Third trimester	34	3.44	11	23	0	1.29(0.32–6.37)
No/unknown	12	1.22	3	9	0	RE
Prenatal examination times			χ2* *=* 8.60, *P* = 0.01			
0/unknown	107	10.84	24	83	0	0.49(0.29–0.82)
1–5	601	60.89	218	372	11	0.99(0.74–1.33)
≥6	278	28.17	102	172	4	RE

* The *χ2* was used to test the difference of epidemiological, delivery, and maternal care characteristics between unavoidable and avoidable maternal deaths. RE was reference group. The OR (95%CI) was used to test the rate ratio of the risk factors between unavoidable and avoidable maternal deaths in the simple logistic regression model.

### Risk factors of unavoidable maternal deaths

The risk factors associated with unavoidable maternal deaths was above 35 years (*aOR* = 1.80 95%*CI*: 1.27–2.55), without prenatal examination (*aOR* = 8.97 95%*CI*:1.11–7.78), low household incomes (*aOR* = 1.15 95%*CI*: 1.02–1.29), without adopting the new deliver way (*aOR* = 5.15 95%*CI*: 3.20–8.31), and death location (*aOR* = 1.09 95%*CI*: 1.02–1.18) (Table 3).

**Table 3:** Factors associated with unavoidable maternal deaths in binary logistic regression model.

Factors	*B*	*P*	aOR*	aOR*(95% CI)	
Age groups (RE: <20 year)		0.003			
20–34 year	0.077	0.878	1.080	0.406	2.872
≥35 year	0.587	0.001	1.798	1.267	2.551
Prenatal examination (RE: ≥6 times)		0.02			
0/unknown	2.194	0.040	8.968	1.105	7.2777
1–5 times	1.608	0.144	4.994	0.579	43.110
Delivery locations (RE: Municipal/provincial hospital)	-0.327	0.000	0.721	0.620	0.840
Death locations (RE: Municipal/provincial hospital)	0.090	0.018	1.094	1.015	1.180
Mode of delivery (RE: Undelivered)	0.310	0.001	1.364	1.135	1.640
Adopt new delivery way (RE: Yes)	1.640	0.000	5.153	3.196	8.309
Educational level (RE: Bachelor or above)	-0.191	0.050	0.826	0.683	1.000
Household incomes per capita(RE:≥8000CNY)	0.138	0.018	1.148	1.024	1.288

* The *aOR* (95%CI) was used to test the rate ratio of the risk factors for unavoidable maternal deaths compared with the avoidable maternal deaths in the binary logistic regression model when adjusted variables of age groups, educational level, household incomes per capita, death locations, delivery locations, the days from delivery to death, parity, gestational age, mode of delivery, adopt new way to deliver, prenatal examination times.

### Review of maternal death

The total number of avoidable deaths was 627 (63.5%), and 345 (35%) cases were judged to be unavoidable, and 15 (1.5%) was agreed to unreviewable for lacking of information. Using the evaluation standards of maternal death review, incorporating the twelve lattice law established by the WHO, this review result showed that the leading risk factor was the knowledge and skills of health care workers in the county/district (*N* = 212, 33.8%), the second risk factor was the knowledge and skills of individuals/families and province/municipal (*N* = 105, 16.7%), and the third was the knowledge and skills of health care workers in the town/village (*N* = 108, 17.2%) (Table 4).

**Table 4:** The number of avoidable maternal deaths judged by the twelve lattice law in Hunan province, China, between 2009 and 2014.

	Individuals/families	Municipal/provincial	County/district	Town/Village	Society departments	In totall
Knowledge and skills	105	105	212	108	0	530
Attitude	17	6	14	12	0	49
Resources	0	2	2	0	0	4
Management	0	7	18	17	2	44
In totall	122	120	246	137	2	627

## Discussion

We found a moderate reduction in the MMR of Hunan province in China from 2009 to 2014. This decrease was especially pronounced in urban areas, compared to that in rural areas. The MMR in Hunan province from 2009 to 2014 was higher than the average MMR in high-income countries (17 per 100,000 live births), but was lower than the MMR reported for Eastern Asia regions, at 27 maternal deaths per 100,000 live births. Compared with the whole of China, the average MMR was 26.23 between 2009 and 2014, with a 22.93% and a 34.71% reduction, respectively, of the MMR in rural and urban areas of China [11, 12]. While Hunan’s decline in MMR had accelerated, it was still higher than the average MMR of eastern areas of China, where the average MMR was 16.22 from 2009 to 2014.

Such a significant reduction in the MMR of Hunan province occurred in the context of China’s national priorities and efforts to improve MCH services in Hunan province. Since 2000, the Chinese government has launched the maternal mortality control project, a project aimed at eliminating neonatal tetanus, and also the subsidy policy for rural women to attend hospital for childbirth was implemented in 378 counties of 12 provinces/autonomous regions/municipalities in central and western China [13]. Hunan’s government has led the efforts to decrease maternal mortality in a number of different ways: by construction of county-level obstetric emergency centers, strengthening the network construction of critical pregnancies, ensuring the flow of the green passage for rescuing the pregnancies smooth, ensuring the resource of obstetric blood sufficient, carrying out the appropriate technical training for copying with obstetric bleeding, promoting the comprehensive prevention and treatment of postpartum hemorrhage in rural areas, making hospital delivery free in rural areas, and creating strategies for improving the knowledge, attitude, and skill of health professionals, such as training classes for rural MCH professionals in Hunan province.

The eastern regional level of economic development and maternal health was better than that of the central and western regions of China with greater territory [5, 14]. The MMR in Hunan province was higher than that in Henan province, with 17.3 in 2009 [7], and Wuhan city in Hubei province, with 10.88 to 10.63 from 2009 to 2012[15]. Jiangsu province in the east of China had the lowest MMR, 1.2 in 2011, which then remained at 1–2 over the next few years [2].

Although the number of direct obstetric deaths decreased in Hunan province between 2013 and 2014, it was still the major cause (50.05%) of maternal deaths. This study showed that pregnancy complication was the leading death cause during the study period, and during the period of 2012 to 2014 based on the annual death rate. Furthermore, while pregnancy complication was the leading cause of death in urban areas, hemorrhage was the leading cause in rural areas. From our results, we can conclude that the proportion of direct obstetric causes in maternal deaths had fallen over time, and that pregnancy complications rather than hemorrhage, had become the leading cause of maternal death, with this phenomenon prominent in urban areas of Hunan province. In determining trends in the causes of maternal deaths, it is reasonable to conclude that the proportion of indirect deaths is increasing in all regions [16]. The selective two-child policy put forward in the Third Plenary Session of the 18^th^ Chinese Communist Party Central Committee in November, 2013, which led to a higher rate of divorce and remarriage, and thus increased the proportion of aged puerpera. However, as age and parity increased, so did the rates of abortion, premature delivery, fetal distress, stillbirth, gestational diabetes mellitus, and other pregnancy complications [17, 18]. The main strategies to reduce MMR are based upon the understanding that pregnancy complications are emergencies and that most deaths occur during a very short period around childbirth. Furthermore, the consequences of maternal morbidity caused by the indirect causes of death can last a long time and lead to further burden.

The study showed above 35 years, low household incomes, without prenatal examination, without adopting the new way to deliver, and death location was associated with unavoidable maternal death, as well as some differences in the distribution of epidemiological, delivery, and maternal care characteristics of maternal death cases. Women at the extreme ends of the reproductive age range (younger than 20 years and older than 35 years) have a higher risk of death for both physiological and social cultural reasons [19]. In this study, approximately 25.1% of all deaths were women who were over 35 years of age, which was similar with data reported for the United States between 2006 and 2010[18]. In our study, most maternal deaths occurred in mothers who attended junior middle school and below, had an annual income per capita below 4000, and resided in the mountains. Low-income groups are the risk factor for maternal deaths. Increased household income and subsidies is likely to provide increased financial access to hospital delivery [20] and therefore reduce delays in care-seeking around the time of birth. This suggested that the government should take targeted measures to alleviate poverty and provide better medical service accessibility.

Nearly 100% of women now give birth in a hospital, but our findings indicated that 20.7% maternal death cases chose municipal or provincial hospital to give birth, where the percentage of maternal deaths was 31.6%. It showed that the provincial/municipal hospital were prone to maternal deaths. The reasons may be that they were the centers of treatment of critical pregnant women. What is more, it had been made progress in rural areas for controlling maternal deaths with the national policy. Therefore, it was not hard to explain that unavoidable deaths would be more likely to occur in the maternal pregnant with higher education levels though most maternal deaths occurred in mothers who attended junior middle school and below. The majority of maternal deaths occurred in last trimester (above 36 gestational weeks or more), with nearly 60% occurring in the first week after birth. Nearly a quarter of deaths occurred within 24 hours of delivery. Our data clearly show that the majority of maternal deaths occurred in the third stage and within 24 hours postpartum. Most maternal complications are emergencies and cannot be predicted with sufficient accuracy [21]. Women with postpartum hemorrhage had less than 2 hours before death; for antepartum hemorrhage, eclampsia, obstructed labor, and sepsis, patients would have 12 hours, 2 days, 3 days, and 6 days, respectively, these timings were agreed by maternal health experts at the 1987 launch of the Safe Motherhood Initiative. Amniotic fluid embolism is a rare complication of pregnancy associated with comparatively high mortality [22]. A Chinese study, investigating patients between 1996 and 2013, showed that the time from the onset of an amniotic fluid embolism episode until death mostly lay within the first 2 hours [23]. However, this highlighted the coverage and quality of care that women receive across the continuum of care, and suggests that we should focus on strengthening the third pregnancy stage and postpartum care for the first 7 days and improving obstetric rescue ability, especially with regards to postpartum hemorrhage, first aid for amniotic fluid embolism, and particularly in improving the ability of midwifery service skills in county hospitals and the obstetric emergency services in provincial/municipal hospitals for critically ill pregnant women in urban areas.

Maternal deaths for single parity and caesarean section were 42.6% and 34.7%, respectively. Since more and more women of advanced age are giving birth under the influence of the Chinese childbearing policy, we are expecting to see more women bearing two births. The population of mothers giving birth is progressively becoming older with substantial impact upon the incidence of caesarean section [24]. In future, this phenomenon will be more distinct under the influence of the universal two-child policy. Although this new policy is unlikely to lead to a significant increase in the mean birth rate for China as a whole, the highly heterogeneous development across the country means that increases in fertility may be greater in particular regions, such as in some rural areas and small towns [25]. The authorities will need to increase the capacity in MCH services, especially in rural areas, based upon monitoring changes in maternal deaths.

The maternal care characteristics showed that nearly 10% of cases were not examined prenatally while 60.9% of cases had one to five prenatal examinations. Early recognition of pregnancy was associated with improved timing and the number of prenatal care visits; the promotion of early pregnancy recognition could therefore represent a means of encouraging and empowering women to access prenatal care at a critical point in fetal development and maternal heath [26]. Pregnancy women in rural areas are advised to attend at least five prenatal care visits, and eight or more times in urban areas.

In this context, avoidable maternal cases accounted for 63.5% of deaths. In order to gain a clearer picture of what role preventability played in maternal deaths, this study identified and categorized the types of preventable events which occurred in the deaths reported. The most frequent and important factors associated with avoidable deaths was improper knowledge/skills of medical institutions in the county, followed by improper individual/family knowledge/skills. In China, the lack of education and technology were probably the major problem underlying poor medical services in rural China [10, 27]. The insufficient knowledge/skills of the health professionals in medical institutions at the township and county level usually arise from a variety of conditions such as a delay in identifying or handling problems or risks during pregnancy, delivery, or puerperium; ignorance of proper referral for certain diseases; and poor surgical skills. Knowledge/skill of the individual/family appeared to be related to the under-educated or ill-informed, resulting in ignorance of pregnancy related risks, absence of prenatal examination, or the inability to identify risks of pregnancy-related complications. The new two-child policy means that increases in fertility may be greater in particular regions, such as in some rural areas and small towns [25]. The acute shortage of pediatricians and pediatric nurses across the country, especially in rural areas, has worsened over the past decade [28], so any increase in birth rate resulting from the two-child policy will exacerbate pressures on an already stressed system in rural areas. This suggests that the provincial health administration department should increase the number of rural health care personnel and strengthen their training.

This study has some limitations. Firstly, workers needed to acquire data within 7 days of death in order to avoid a large amount of data missing. Secondly, there was information bias when the workers of count/district MCH investigated the situation of maternal deaths, such as recall bias, survey bias. Thirdly, the resident population included permanent and temporary residents (with at least one year of residence) in the Hunan PMMSS. However, China has experienced a large migration from rural to urban area, or from Hunan province to developed provinces and cities, such as Guangdong province. Migrant women were registered as rural inhabitants, but shared urban prenatal health care, or permanent residents shared better prenatal health care in other provinces.

## Conclusions

Moderate progress has been made in reducing the MMR of Hunan province. We propose that the government focus its efforts on the following aspects in future. The first is to improve basic midwifery skills in rural areas, thus guaranteeing that each pregnant woman will receive at least five high-quality prenatal examinations, to improve the obstetric emergency rescue service ability in urban areas for critically ill pregnant women, and to guarantee that each pregnant woman will receive at least eight high-quality prenatal examinations. The second is to strengthen maternal mortality monitoring across the whole province, increase the number of rural health care personnel, and strengthen their training to improve knowledge/skills of the health professionals in medical institutions, especially given that the future influence of the universal two-child policy.
